# Disulfiram-Induced Acute Liver Injury

**DOI:** 10.1155/2020/8835647

**Published:** 2020-09-09

**Authors:** Lucas Ramer, Matthieu Tihy, Nicolas Goossens, Jean-Louis Frossard, Laura Rubbia-Brandt, Laurent Spahr

**Affiliations:** ^1^Gastroenterology and Hepatology, University Hospitals of Geneva and Faculty of Medicine, Geneva, Switzerland; ^2^Clinical Pathology, University Hospitals of Geneva and Faculty of Medicine, Geneva, Switzerland

## Abstract

Disulfiram is a drug used to treat alcohol dependence since many years. It interferes with the metabolism of alcohol, may be associated with neurological and dermatological symptoms, and can be hepatotoxic. Due to the frequent coexistent liver test alterations due to alcohol, the true incidence of disulfiram-associated liver injury is unclear and severity of injury may vary from mildly elevated liver enzymes to fulminant hepatitis leading to death. There are several reported cases of disulfiram hepatitis in the literature. Liver histology, when available, demonstrates some degree of portal inflammation with eosinophils and hepatocyte necrosis. We present here a well-documented case of acute hepatitis due to disulfiram with typical histological lesions, favorable outcome following drug withdrawal, and a brief steroid course. The risk of hepatotoxicity should be kept in mind when prescribing disulfiram.

## 1. Introduction

Disulfiram is a drug prescribed in many countries as a supportive treatment for alcohol dependence, by generating unpleasant symptoms when drinking alcohol [1]. Accordingly, the mechanism of action of the drug is based on the inhibition of acetaldehyde dehydrogenase, a key enzyme in alcohol metabolism. Consequently, acetaldehyde accumulates in blood and induces symptoms including headache, flushing, nausea, and vomiting. Side effects of disulfiram include fatigue, abdominal discomfort, and sometimes serious dermatological, neurological or psychiatric manifestations [2]. Although considered a relatively safe drug from an hepatological point of view, cases of disulfiram-associated liver injury have been reported several years ago [3–7], sometimes presenting as a fulminant hepatic failure [8, 9]. Available data favor an idiosyncratic reaction occurring after several weeks of exposition, with a positive rechallenge and a causal relationship established in most cases [10]. However, description of liver injury in a biopsy is inconstant, and histological alterations are sometimes difficult to characterize due to a coexistent advanced alcoholic liver disease [3]. Thus, we present here in detail the case of an acute disulfiram-induced hepatitis including a detailed description of histological findings on a liver biopsy and a full recovery following a brief steroid treatment.

## 2. Case Presentation

**Figure 1:**
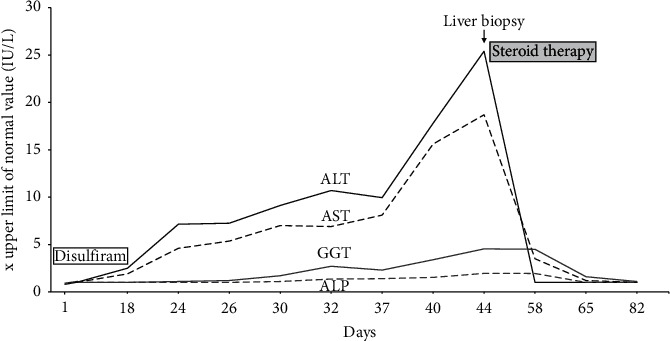
Graphical illustration of the course of disulfiram-induced acute hepatitis. Biological tests (AST: aspartate aminotransferase; ALT: alanine aminotransferase; GGT: gamma-glutamyl transferase; ALP: alkaline phosphatase) given as upper limit of normal value.

**Figure 2:**
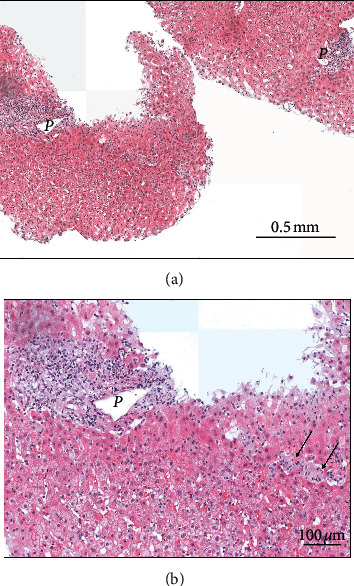
Histological view of the liver biopsy specimen (hematoxylin-eosin stain) showing normal architecture and no fibrosis (original magnification ×100, panel A). At a closer view (panel B, original magnification ×200), an inflammatory infiltrate is present in the portal tract P, predominantly lymphocytic, associated with scattered eosinophils. In the liver lobule, few necrotic hepatocytes and occasional microgranulomas (arrows) are visible. Periodic acid Schiff (PAS) and Ziehl staining are negative for infections.

A 47-year-old woman was referred to our department on February 9, 2020, for abnormal liver function tests. She has no relevant medical history except for an alcohol use disorder detected 3 years ago for which she was referred to an addiction specialist in mid-2019. At this time, she met the criteria for alcohol dependence and was initially managed by a combination of psychosocial support and benzodiazepines. As this strategy failed to control the ongoing alcohol misuse, pharmacotherapy using an anticraving agent was decided. At that time, liver transaminases were normal. Thus, disulfiram was started on December 18, 2019, at 200 mg daily, corresponding to half the recommended dose [2]. During the first ten days, the drug was well tolerated at this reduced dose and the patient did not declare any alcohol consumption during this period. On December 29, the posology was further reduced to 200 mg every other day due to apparition of nausea, fatigue, and abdominal discomfort. These symptoms were considered in relation with disulfiram therapy, and serum alcohol levels were not monitored. Laboratory tests 18 days after treatment initiation revealed an elevation of AST (91 IU/L (*N* : <50)) and ALT (127 IU/L (*N* : <50) , while gamma-glutamyl transferase (GGT), alkaline phosphatase (ALP) and serum bilirubin were normal. Disulfiram was then stopped. At physical examination, the patient had no signs of preexisting liver disease. At abdominal echography, the liver was slightly hyperechoic without signs of chronic liver disease. Additional blood tests showed normal INR, hemoglobin, platelets, and white blood cells with normal eosinophil count. Total IgG levels were in the normal range, while antismooth muscle and anti-actin antibodies were negative. The patient was, also, negative for hepatitis A, B, C, and E. Ceruloplasmin levels were in the normal range. She refused coadministration with other medications or illicit drugs and did not present skin rash, headache, or fever. Since disulfiram was stopped, transaminases continued to increase, as well as GGT and ALP to a lesser extent. At day 44, after initiation of disulfiram, values of ALT and AST reached 1526 IU/L and 934 IU/L, respectively, while serum bilirubin remained normal. In order to determine more precisely the etiology and severity of this acute hepatitis, we performed a liver biopsy that showed portal infiltration with lymphocytes and eosinophils, mild biliary tract dystrophy, and scattered necrotic hepatocytes in the liver lobule together with microgranulomas. There was no steatosis and no fibrosis. A diagnosis of acute drug-induced liver injury (DILI) due to disulfiram was made, as histological lesions were not consistent with alcoholic liver injury, and an alternative etiology was reasonable excluded.  Figure 1 illustrates the clinical course of the hepatitis and associated biological alterations.  Figure 2 illustrates histological lesions on liver biopsy.

Consistent with previously reported cases of disulfiram hepatitis [10], biological course was suggestive of an idiosyncratic reaction. Although there are no firm recommendations for steroids in acute DILI [11], we decided to initiate prednisone at a dose of 30 mg daily with gradual tapering off the dose, which was associated with a rapid diminution of transaminases and a return to completely normal values after 20 days of treatment. A strong recommendation was made to avoid reexposition to this aversion therapy.

## 3. Discussion

Acute liver injury due to disulfiram (tetraethylthiuram disulfide) is relatively uncommon and typically develops within 2 to 12 weeks following initiation of treatment [2, 10, 12]. The risk of severe hepatitis with liver insufficiency leading to death or liver transplantation is even less frequent, with an estimation of 1 fatal case in 30,000 treated patients per year [2]. This risk seems unrelated to the presence of an underlying liver disease, is not dose-related, and is thought to result from a hypersensitivity reaction involving P450 cytochrome enzymes [2, 11]. Using data from a Swedish registry of DILI, Björnsson et al. [10] identified 82 reports of disulfiram-induced liver injury over a 36-year period, corresponding to 1 per 1.3 million average daily dose of the drug. In Denmark, over a 5-year period, disulfiram hepatitis was the second most frequent cause of referral to a liver transplant center [13]. The true incidence of disulfiram-induced DILI is difficult to determine in a population of patients with frequent biological and histological alterations due to alcohol misuse. In alcoholic steatohepatitis, the rise in transaminases never exceeds six times the upper limit of normal value [14], while acute alcoholic microvesicular steatosis typically manifests with more elevated liver enzymes [15]. Nevertheless, when acute DILI is suspected, the pattern of injury based on the *R* ratio calculation of liver biological tests is useful [11]. The hepatocellular pattern of injury is more frequently reported as compared to the cholestatic pattern. To attribute liver injury to a particular drug needs to consider many items grouped under a scoring system to assess causality [16]. When using this scale in our patient, the responsibility of disulfiram in the occurrence of acute hepatitis is determined as “probable.” The absence of extrahepatic manifestations including eosinophilia and no reexposition to the drug prevented to reach the state of a “definite” causal relationship.

A liver biopsy provides major information that may help to confirm, or infirm, a diagnosis of DILI. Based on reported cases, disulfiram-associated lesions include the following: portal tract inflammation mostly composed of mononuclear cells and eosinophils, mild bile ducts alterations, and necrotic and apoptotic hepatocytes in the liver lobule. These lesions were observed in our patient, together with a microgranulomatous pattern of lobular inflammation, which has not been reported in previous histological reports.

In our patient, we stopped disulfiram and initiated tapered doses of steroids in order to shorten recovery time. Corticosteroid therapy in DILI remains controversial [17], and the decision to treat depends mostly on the physician's personal experience.

Although disulfiram is not recognized nowadays as a major tool to treat alcohol dependence [18], it is described as a relatively safe treatment with no liver-related deaths reported in recent years [2, 18]. Nevertheless, the risk of hepatotoxicity needs to be kept in mind and transaminases need to be regularly monitored when starting a treatment with disulfiram. The typical pattern of injury is hepatocellular, and the fatality rate may reach 10 to 15% if jaundice complicates the course of such DILI [13]. In case of any doubt about the diagnosis of disulfiram-induced liver injury, a liver biopsy may help to confirm the diagnosis by excluding alternative causes of acute injury including alcoholic liver disease.

To conclude, we present here in detail a case of acute drug-induced liver injury resulting from a brief exposition to disulfiram, with a pattern of hepatocellular damage and a mechanism consistent with drug hypersensitivity.

## Abbreviations

DILI: Drug-induced liver injury
AST: Aspartate aminotransferase
ALT: Alanine aminotransferase
GGT: Gamma-glutamyl transferase
ALP: Alkaline phosphatase.
